# The Physician in Art

**Published:** 1908-01-11

**Authors:** 


### The Physician in Art.

The great and little writers, with few exceptions, have treated the medical profession badly, and he who wishes to compile from the writings of both a book to the detriment of the doctor will find much material at hand. We do not say that the field for him who attempts to arrange a volume of extracts on the other side will not be ample. There are excellent doctors in fiction, and lovers of good books will doubtless be able to point to one or two medical characters in their favourites which are realistic portraits, and not mere caricatures. Douglas Jerrold, now so rarely read, drew them, and the military surgeons in Samarow's romances will be familiar enough to students of German literature. The pæan in praise of the doctor has been well sung by several poets, and if Crabbe, himself a wielder of the lancet, has left on record the Hogarthian delineation of the village doctor—a sketch that revolts even yet by its callousness—Meredith has earned the gratitude of the honest physician by singing the virtues of Melampus. In literature the physician is well represented, and it is interesting to see what he has been to the painter and sculptor.

There have been few attempts to criticise the painter's physician from the professional standpoint, and with the exception of Holländer's well-known work dealing with the influence of medicine upon classical art (a classic in itself which we should like to see translated and brought more prominently to the notice of the profession) there has been as few attempts to study the artist's conception of the doctor from a general aspect. For that reason Dr. John Churchman's article "The Physician in the Paintings of Jan Steen," which appears in the current issue of the Johns Hopkins Hospital "Bulletin" is of more than ordinary interest, for it is not merely a chatty résumé of the painter's work and life, but it is an endeavour to estimate what light his works throw on the doctor of his day and on the latter's standing in the social, his place in the intellectual life of the times. To untravelled Englishmen Jan Steen is a little-known Dutch artist, represented in the national collection by two small pictures, the "Terrace Scene" and the "Music Lesson." To those, however, who have not been able to view for themselves the excellent examples of the master's work preserved in the Amsterdam Ryks Museum, at The Hague, Rotterdam, and Brussels, the beautiful photographic reproductions which illustrate Dr. Churchman's article will come as a surprise, and to those who are interested in art and its relation to the profession we cordially commend the reading of Dr. Churchman's sympathetic exposition of these types. With his conclusions everyone who is acquainted with Dutch art must thoroughly agree. There is little that is ridiculous in Jan Steen's representations of the doctors of his time: there is much that is grandly superb in the conceptions which his contemporaries who wielded the brush formed of the physicians of the day. Witness the wonderful "Anatomische Les" and "The Dissection of a Head" by Rembrand, the "Voorlesing in Ontleedkunde" of de Keyser, and the similar, though differently treated subjects of Van Neck and his school. Nor when we come to other painters do we find that they have treated the physician less chivalrously. Titian's Parmese doctor and Holbein's portrait are examples in point. Excluding mere portraits, it is safe to say that the physician in art has usually been represented as a subject that appeals. Even the quacks have been dignified, as if the artists rigidly followed old Chrome's dying injunction to his son. As Dr. Churchman justly concludes, "The message of the Dutch painters is that as a matter of fact, and on the average, medicine in the seventeenth century did appeal to men whose intellects, manners, and bearing are worth study." And the verdict is one that must apply to all art, except where the artist has honestly set himself to caricature instead of faithfully interpreting what he saw.

